# Risk Factors and Causes of Inpatient Falls in a Tertiary Care Hospital in Tehran, Iran; A Retrospective Study

**DOI:** 10.34172/aim.33365

**Published:** 2025-07-01

**Authors:** Maryam Ameri, Foroozan Faress, Siamak Soltani, Abbas Aghabiklooei, Leyla Abdolkarimi, Amirmohammad Babaeigoharrizi

**Affiliations:** ^1^Department of Forensic Medicine, School of Medicine, Iran University of Medical Sciences, Tehran, Iran; ^2^Rajaie Cardiovascular Medical and Research center, Tehran, Iran; ^3^Department of Orthopedic Surgery, Iran University of Medical Sciences, Tehran, Iran

**Keywords:** Accidental falls, Clinical safety, Falls prevention, Hospital falls, Inpatient falls

## Abstract

Inpatient falls are a significant health concern, leading to increased hospital stays, costs, and potential severe injuries or death. This study aims to investigate the incidence, risk factors, and challenges of inpatient falls in Firoozgar hospital. This cross-sectional study was conducted in a tertiary care hospital in Tehran, Iran. The study included all patients who experienced a fall incident in Firoozgar hospital between April 2023 and March 2024. We included 90 patients with an average age of 57.49 years. Falls were more prevalent among men, older patients, and individuals with lower education levels. Underlying conditions such as diabetes, heart disease, and addiction significantly increased the risk of falls. Identifying the specific causes of falls in each hospital ward can facilitate the development of targeted preventive strategies. Implementing environmental modifications and fall prevention measures could substantially reduce fall risks, enhance patient safety, and lower healthcare costs.

## Introduction

 Falls are recognized as a major healthcare concern among hospitalized patients, lead to prolonged hospital stays and increased healthcare costs, and may result in severe injuries or even death.^1^ Beyond physical impacts, falls can also lead to psychological distress and social consequences such as loss of confidence, fear of falling, activity avoidance and low self-efficacy.^2^

 In the United States, reported hospitals fall rates range from 3.3 to 11.5 falls per 1000 patient-days, although the true incidence may be higher due to underreporting of fall events.^3^ In Iran, a cross-sectional study conducted between 2017 and 2019 on patients in two central hospitals in Qazvin city reported a fall incidence of 2.5 per 1000 patient-days.^4^

 Falls are particularly more prevalent in older adults, who are considered a vulnerable population.^5^ With advancing age, the rate of falls and its consequences also increase. Fall rates in geriatric wards are as high as 20 falls per 1000 patient-days, and the incidence of falls is generally higher in hospital settings compared to community environments. Moreover, more than 85% of all fall-related mortality occurs in people aged over 65.^5^ In Iran, several factors have been identified to increase the risk of inpatient falls, including longer hospital stays, use of chemotherapy drugs, sedatives, anticonvulsants, and benzodiazepines, visual impairment, balance disorders, manual transfer aids, and urinary incontinence.^6^

 The direct costs of fall-related injuries vary, accounting for 0.1% to 1.5% of total healthcare expenditures in the U.S. and European countries.^7^ In the USA, the costs were estimated at US$ 616.5 million for fatal falls and US$ 30.3 billion for non-fatal fall injuries in 2012. Furthermore, patient falls in hospitals result in extended hospital stays, which also increases the likelihood of subsequent falls, further increasing healthcare costs.

 Given the high incidence of falls among hospitalized patients, their significant financial and physical burdens, and the limited studies conducted in Iran compared to other countries, this study aims to assess the incidence of falls and identify risk factors.

## Materials and Methods

 This cross-sectional study was conducted in a tertiary care hospital in Tehran, Iran. The study included all patients who experienced a fall incident in Firoozgar hospital, Tehran, between April 2023 and March 2024. Inclusion criteria were hospital admission to Firoozgar hospital during the specified period and documentation of a fall during hospitalization. Exclusion criteria included falls occurring outside the hospital and insufficient documentation of the in-hospital fall event.

 Data were extracted from hospital fall event reports, which were documented in the patient safety division of Firoozgar hospital. Information such as age, gender, underlying medical conditions, reason for hospitalization, ward where the fall occurred, medications received during hospitalization, use of assistive devices, and physical or psychological injuries sustained from the fall was retrieved from the hospital’s electronic records. Data extraction was performed by an expert who serves as the head of the hospital’s safety division.

 Missing data were addressed by excluding incomplete records from the analysis. To ensure patient confidentiality, data were anonymized by replacing patient identifiers with unique codes, and only the data extractor had access to identifiable information. As this was a retrospective study, patient consent was waived in accordance with institutional policies and ethical guidelines. The study received ethical approval from the Ethics Committee of Iran University of Medical Sciences (Ethics Code: IR.IUMS.FMD.REC.1400.608).

## Results

###  Patient Demographics

 A total of 90 patients hospitalized in Firoozgar hospital who experienced a fall during their stay were included in the study. The mean age of the patients was 57.49 + 2.75 years, ranging from 21 to 86 years. Of these, 34 patients (37.8%) were younger than 57 years, and 56 patients (62.2%) were 57 years or older. Falls occurred more frequently among male patients, with 64 cases (71.1%) compared to 26 cases (28.9%) in female patients.

Table 1 demonstrates the demographic features of patients, risk factors, and causes of falls.

**Table 1:** Demographic Characteristics of Patients, Along with Detailed Distribution of Risk Factors and Causes of Falls

**Characteristics**	**N=90**
Demographics	
Mean age ( + SD)	57.49 ( + 2.75)
Female, *n* (%)	26(28.9%)
Educational background	
Illiterate	19 (21.1%)
Elementary education	15 (16.7%)
High school diploma	33 (36.7%)
Fall incidence by hospital ward	
Infectious disease	13 cases (14.4%)
ICU	10 (11.1%)
Emergency departments	10 (11.1%)
Hematology and oncology ward	9 (10.0%)
Other departments	8 (8.9%)
Causes of Falls	
Falling off a bed	58 (64.4%)
Falling from the bathroom	17 (18.9%)
Falling on an even surface	7 (7.7%)
Slipping	4 (4.4%)
Falling off a wheelchair	3 (3.3%)
Falling in the shower	1 (1.1%)
Fall incidence by time of day and night shift	
Falls during the day shift (7 AM to 7 PM)	48 cases (53.3%)
Falls during the night shift (7 PM to 7 AM)	42 cases (46.7%)
Fall incidence by hospitalization day	
First day	13 (14.4%)
Second day	18 (20.0%)
Third day	14 (15.6%)
Fourth day	10(11.1%)
Fifth day	7 (7.8%)
Sixth day	6 (6.7%)
≥ Seventh day	22(24.4%)
Underlying medical conditions	
Diabetes	33 (36.6%)
Hypertension	30 (33.3%)
Cardiovascular disease	23 (25.5%)
Mental health conditions	16 (17.8%)
Cancer	10 (11.15)
Neurologic disease	10 (11.15)
Mental health conditions	
Generalized anxiety disorder	8(8.9%)
Major depressive disorder	5(5.6%)
Alzheimer's disease	2(2.3%)
Bipolar disorder type 1	1(1.2%)
Drug and alcohol abuse	
Opioid	15 (16.7%)
Methamphetamine	1(1.1%)
Alcohol	5 (5.6%)
Use of mobility aids	
Wheelchair	11 (12.2%)
Cane	4 (4.4%)
Walker	3 (3.3%)
Crutches	1 (1.1%)
Vision status	
Normal vision	70 (77.8%)
Using prescription glasses	11 (12.2%)
Blurry vision	6 (6.7%)
History of cataract surgery	2 (2.2%)
Blindness in the left eye	1 (1.1%)

###  Educational Background

 Among the study participants, 33 patients (36.7%) had a high school diploma, 19 patients (21.1%) were illiterate, and 15 patients (16.7%) had an elementary education.

###  Fall Incidence by Hospital Ward

 Of the 90 fall incidents, 13 cases (14.4%) occurred in the infectious disease ward, 10 cases each (11.1%) in the ICU and emergency departments, 9 cases (10.0%) in the hematology and oncology ward, and 8 cases (8.9%) in other departments.

###  Causes of Falls

 The primary causes of falls were as follows: 58 cases (64.4%) involved falling off a bed, 17 cases (18.9%) falling in the bathroom, 7 cases (7.7%) on an even surface, 4 cases (4.4%) by slipping, 3 cases (3.3%) off a wheelchair, and one case (1.1%) falling in the shower.

###  Fall Incidence by Time of Day and Night Shift

 Falls occurred in 48 cases (53.3%) during the day shift (7 AM to 7 PM) and 42 cases (46.7%) during the night shift (7 PM to 7 AM). The highest frequency of falls occurred during the night from 7 PM to 4 AM (35 cases).

###  Fall Incidence by Month

 The highest incidence of falls was observed in June with 11 cases (12.2%), followed by August and April, with 10 cases each (11.1%).

###  Fall Incidence by Hospitalization Day

 Falls occurred on the second day of hospitalization in 18 cases (20.0%), the third day in 14 cases (15.6%), and the first day in 13 cases (14.4%). In total, 61.1% of falls occurred within the first four days of hospitalization. Additionally, all patients (100%) had an intravenous (IV) line placed upon admission, which was present at the time of the fall.

###  Underlying Medical Conditions

 Seventy-one patients (78.8%) had underlying medical conditions. The most common were diabetes (33 patients, 36.6%), hypertension (30 patients, 33.3%), and cardiovascular disease (23 patients, 25.5%).

###  Mental Health Conditions

 Among the patients who experienced a fall, eight patients (8.9%) were being treated for generalized anxiety disorder (GAD), five patients (5.6%) for major depressive disorder (MDD), two patients for Alzheimer’s disease, and one patient for bipolar disorder type 1 (B1D).

###  Sedative and Medication Use

 Thirty-eight patients (42.2%) had taken sedative medications on the day of the fall. The most common medications were benzodiazepines (12 cases, 13.3%), Depakine (8 cases, 8.9%), and Levebel (7 cases, 7.7%).

###  Substance use History

 Among the 90 patients, 74 (82.2%) reported no history of substance abuse. However, 15 patients (16.7%) had a history of opioid use, and one patient (1.1%) had a history of methamphetamine use.

###  Alcohol Consumption

 Of the patients who experienced a fall, five patients (5.6%) reported alcohol consumption, while 85 patients (94.4%) had no history of alcohol use.

###  Use of Mobility Aids

 Seventy-one patients (78.9%) did not routinely use mobility aids, while 11 patients (12.2%) used a wheelchair, 4 patients (4.4%) used a cane, 3 patients used a walker, and one patient used crutches.

###  Vision Status

 In terms of vision status, 70 patients (77.8%) had normal vision, 11 patients (12.2%) used prescription glasses, 6 patients (6.7%) reported blurry vision, 2 had a history of cataract surgery, and one patient was blind in the left eye.

## Discussion

 In this study, we identified that the highest incidence of falls in a tertiary care hospital in Tehran occurred among individuals aged 60 to 65 years, with a higher frequency of falls observed in men compared to women. These findings are consistent with studies by Shali et al and Aghakhani et al, which reported that most falls in hospitals affiliated with Tehran University of Medical Sciences occurred in men and in individuals over 60 years of age.^2^ This alignment emphasizes the fact that demographic factors, particularly age and gender, play a significant role in the occurrence of falls.^2^

 The majority of falls in our hospitalized patients were off the bed (64.4%) and in the bathroom (18.9%). These findings suggest that targeted preventive measures, such as raising bed rails and ensuring the safety and non-slip conditions of bathroom areas, should be considered.

 Soltani et al demonstrated that the highest death rate of ICU patients occured during the night, emphasizing the importance of patient care during this time.^8^

 Interestingly, the second day of hospitalization saw the highest incidence of falls (20%), which is consistent with the study by Fallahi et al, who found that the majority of falls occurred within the first 1–4 days of admission.^9^ These results demonstrate the importance of thorough patient assessment during the early days of hospitalization to mitigate fall risk.

 More than half of the patients who experienced falls had underlying conditions such as diabetes, hypertension, or cardiovascular diseases. These findings emphasize the need for increased vigilance and preventive measures for patients with chronic comorbidities, as they are at greater risk of adverse events, including falls, during hospitalization.

 The use of sedatives and hypnotics, such as benzodiazepines, was associated with a significant increase in fall risk. This observation aligns with previous research, which demonstrated that the use of such medications impairs patient alertness and heightens the likelihood of falls. For instance, Noh et al found that anticonvulsants and benzodiazepines were associated with a high risk of falls in acutely hospitalized elderly patients.^10^ Similarly, Chan et al showed that benzodiazepine use increased fall risk in psychiatric hospitalized patients.^11^

 One-sixth of our patients had a history of opioid use. This is in line with findings by Alexander et al, who identified substance and alcohol use as a fall risk due to its impact on cognitive and motor function.^12^ These findings emphasize that drug and alcohol use contribute to fall risk in some patients.

 Additionally, 21% of the patients who experienced falls were using assistive devices, with wheelchairs being the most common. Impaired vision was another identified risk factor, affecting 20 patients, including those with conditions such as blurred vision, monocular blindness, history of cataract surgery, or the need for corrective eyewear. These findings support previous research indicating that visual impairments and using assistive devices are important risk factors for falls among hospitalized patients.^12^

## Conclusion

 In conclusion, this study has identified multiple risk factors for falls in hospitalized patients, including age, gender, use of sedatives, and environmental factors. To reduce the incidence of falls, targeted preventive strategies are essential, particularly for older adults, patients with unstable conditions, and those on sedative medications. Proactive risk assessments and interventions during the early days of hospitalization, along with environmental modifications, could significantly reduce the risk of falls.

## Limitations

 This study has several limitations. First, it is a descriptive study, and no statistical analysis was conducted to identify correlations or calculate odds ratios for the identified risk factors. Second, we did not calculate the fall incidence rate, limiting our ability to contextualize the data relative to patient volume. Third, data extraction was performed by a single trained individual, and double-checking of the data was not conducted, which may have introduced errors. While the data were extracted from a typically well-documented and standardized incident reporting system, some variables—such as specific medication usage or assistive device usage—might not have been consistently recorded, potentially affecting the completeness and accuracy of the dataset. Future studies should aim to address these limitations by employing more rigorous data collection and statistical methodologies to better understand the relationships between risk factors and fall outcomes.
